# Creation of Dystrophin Expressing Chimeric Cells of Myoblast Origin as a Novel Stem Cell Based Therapy for Duchenne Muscular Dystrophy

**DOI:** 10.1007/s12015-017-9792-7

**Published:** 2018-01-05

**Authors:** M. Siemionow, J. Cwykiel, A. Heydemann, J. Garcia-Martinez, K. Siemionow, E. Szilagyi

**Affiliations:** 1 Department of Surgery, Poznan University of Medical Sciences, Poznan, Poland; 2 Department of Orthopedics, University of Illinois at Chicago, Chicago, IL USA; 3 Department of Physiology, University of Illinois at Chicago, Chicago, IL USA

**Keywords:** Duchenne muscular dystrophy, Stem cells, Myoblasts, Therapy, Mdx mice, Dystrophin, Ex vivo fusion

## Abstract

Over the past decade different stem cell (SC) based approaches were tested to treat Duchenne Muscular Dystrophy (DMD), a lethal X-linked disorder caused by mutations in dystrophin gene. Despite research efforts, there is no curative therapy for DMD. Allogeneic SC therapies aim to restore dystrophin in the affected muscles; however, they are challenged by rejection and limited engraftment. Thus, there is a need to develop new more efficacious SC therapies. Chimeric Cells (CC), created via ex vivo fusion of donor and recipient cells, represent a promising therapeutic option for tissue regeneration and Vascularized Composite Allotransplantation (VCA) due to tolerogenic properties that eliminate the need for lifelong immunosuppression. This proof of concept study tested feasibility of myoblast fusion for Dystrophin Expressing. Chimeric Cell (DEC) therapy through in vitro characterization and in vivo assessment of engraftment, survival, and efficacy in the *mdx* mouse model of DMD. Murine DEC were created via ex vivo fusion of normal (*snj)* and dystrophin–deficient (*mdx)* myoblasts using polyethylene glycol. Efficacy of myoblast fusion was confirmed by flow cytometry and dystrophin immunostaining, while proliferative and myogenic differentiation capacity of DEC were assessed in vitro. Therapeutic effect after DEC transplant (0.5 × 10^6^) into the gastrocnemius muscle (GM) of *mdx* mice was assessed by muscle functional tests. At 30 days post-transplant dystrophin expression in GM of injected *mdx* mice increased to 37.27 ± 12.1% and correlated with improvement of muscle strength and function. Our study confirmed feasibility and efficacy of DEC therapy and represents a novel SC based approach for treatment of muscular dystrophies.

## Introduction

Duchenne Muscular Dystrophy (DMD) is the most frequent and severe form of muscular dystrophy that affects approximately 1 in 4000 newborn boys [1]. It is a genetic, X-linked, progressive disease, characterized by muscle wasting and weakness, which leads to loss of motor function and in consequence to respiratory and cardiac failure resulting in premature death [1]. DMD is caused by mutation of the dystrophin gene leading to a lack of dystrophin which is an essential structural musculoskeletal protein. Despite extensive research efforts and strategies, there is no therapy for DMD and current management consists of supportive and/or palliative efforts. Thus, the major focus of the ongoing research is to develop new strategies which will either cure or slow the progression of DMD. There are several pre-clinical and clinical approaches testing the efficacy of new therapies in DMD, including exon skipping, gene editing via viral vectors [2], and gene slicing CRISPR system [3, 4] delivered by adeno-associated viruses. The clinical efficacy of these therapies is not yet confirmed and the potential long-term side effects of off-site mutations caused by these virus based therapies are of concern. An alternative approach includes stem cell based therapies which are considered by many to be the most promising methods for treatment of muscular dystrophies [5]. Stem cell transplants are based on delivery of either autologous or allogenic stem cells [6–10]. Several cell lines were tested in the clinical scenario as well as in small and large animal models of DMD [11]. Autologous stem cells from DMD patients include bone marrow [12] and mesenchymal stem cell [11] transplants as well as patient’s myoblasts which are genetically altered ex vivo in order to deliver functional dystrophin to the affected muscles [13]. Allogeneic stem cell transplantation of satellite cells [14], mesenchymal stem cells [15] adipose mesenchymal stem cells [16] bone marrow [12], pericytes [17] and iPS [18] confirmed dystrophin expression in small and large animal models of DMD [16]. Despite encouraging early post-transplant results, the long-term outcomes were inconsistent among different investigators. The major problems encountered by all investigators were either limited or short-term cell engraftment [5, 19] and allogenic immune response [20]. Even with the use of immunosuppressive therapy [21], the efficacy of engraftment was sub-optimal and not consistent among published studies and the potential side effects of immunosuppression were of a significant concern [9]. In order to facilitate and maintain long-term engraftment after stem cell transplantation new approaches are needed. To address this need, we propose to introduce the concept of myoblast cell fusion based on our experience with tolerogenic effects of chimeric cell (CC) therapy created by ex vivo stem cell fusion of bone marrow-derived hematopoietic stem cells from the donor and the recipient of vascularized composite allograft (VCA) [22–26]. This approach resulted in the development of donor-specific chimerism and long-term allograft survival. Encouraged by maintenance of engraftment and tolerogenic effects of hematopoietic CC after fusion, we have tested the same approach for myoblast cell fusion between normal and dystrophin -deficient myoblast donors in the *mdx* mouse model of DMD. Here, we present our results of the feasibility of Dystrophin Expressing Chimeric Cell (DEC) creation via ex vivo polyethylene glycol (PEG) fusion technique and assess both in vitro and in vivo dystrophin expression after cell fusion. We confirm significant improvement in muscle strength and function after transplantation of DEC into gastrocnemius muscles of *mdx* mice.

## Materials and Methods

### Experimental Animals

Animal care and experimental protocols were approved by the University of Illinois at Chicago Institutional Animal Care and Use Committee (IACUC). Six to eight -week old mice - *mdx* (C57BL/10ScSn-Dmdmdx/J, stock number 001801) with the respective background *snj* wild type (*wt*) mice (C57BL/10ScSnJ, stock number 000476) were purchased from Jackson Laboratories. Mice were housed in the Molecular Biology Research Building, an AAALAC-accredited animal facility, at University of Illinois at Chicago.

### Ex vivo Creation of Murine Dystrophin Expressing Chimeric Cell (DEC)

#### Primary Myoblast Isolation and Culture from *wt* and *mdx* Mice

Primary murine myoblasts cells were isolated from 10 *mdx* and 10 *snj* wild type (*wt*) mice. Briefly, hind limb muscle was removed. Using sterile surgical blades and forceps, muscle tissue was dissected from bones and placed in DPBS on ice. Muscle tissue was then minced to 1–2 mm pieces and incubated with 1.5U/l Collagenase type D (Roche –ThermoFischer, Waltham, MA, USA) and 2.4U/l Dispase II (Sigma, St. Louis, MO, USA) in 2.5 mM CaCl_2_ solution at 37 °C for 30 min. Using Primary Culture Media (F-10+), Ham’s F-10 medium (Gibco-ThermoFischer, Waltham, MA, USA) supplemented with 20% Fetal Bovine Serum (Gemini Bio-Products, West Sacrament, CA, USA), 1X Antibiotic-Antimicotic solution (Gibco- ThermoFischer, Waltham, MA, USA) and 50 μl of 25 μg/ml basic fibroblast growth factor (bFGF, Peprotech, Rocky Hill, NJ, USA), samples were suspended and digested tissue was further mechanically dissociated by pipetting. Tissue homogenate was filtered through 100 μm and subsequently 70 μm pore size nylon meshes (ThermoFischer, Waltham, MA, USA). The sample was centrifuged (350 g, 5 min) and washed twice with F-10-based primary culture media. After centrifugation, cells were counted and plated in 75 cm^2^ collagen-coated flasks (Celltreat Scientific Product, Pepperell, MA, USA). Upon 70–80% of confluence, adherent cells were harvested with mechanical agitation in DPBS and pre-plated for 15 min in collagen-coated flask to eliminate fibroblasts from culture. Non-adherent cells were transferred for further culture in F-10-based Primary Culture Media. After 3–4 additional passage and pre-plating steps, myoblast were expanded in F10/DMEM-based Myoblast Growth Medium supplemented with 20% Fetal Bovine Serum (Gemini Bio-products, West Sacramento, CA, USA), 1X Antibiotic-Antimicotic solution (Gibco- ThermoFischer, Waltham, MA, USA) and 50 μl of 25 μg/ml basic fibroblast growth factor (bFGF, Peprotech). During expansion, myoblast (MB) harvests were performed with 0.25% Trypsin–EDTA solution (Gibco- ThermoFischer, Waltham, MA, USA). Murine MB, passages 5–7, were used for fusion procedure.

#### Cell Fusion Procedure

**Fig. 1:**
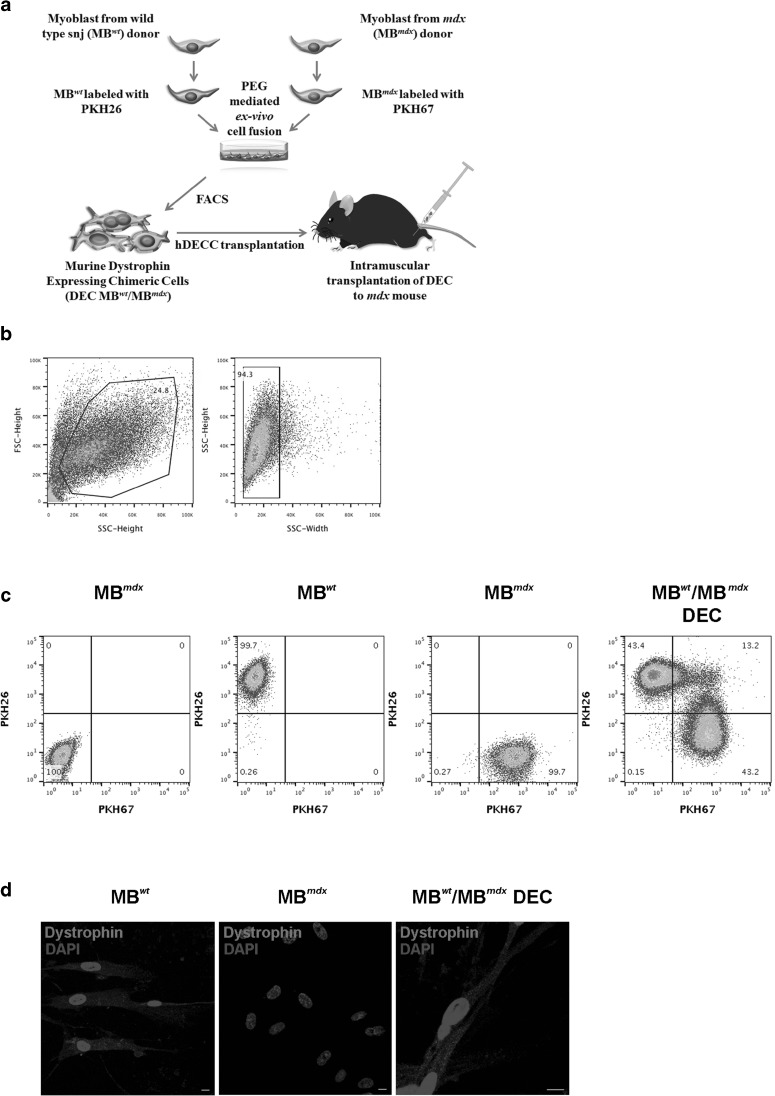
**Confirmation of ex vivo creation of murine Dystrophin Expressing Chimeric Cell (DEC) derived from the wild type** ***snj*** **myoblasts (MB**^***wt***^**) and dystrophin-deficient** ***mdx*** **(MB**^***mdx***^**) mice and confirmation of dystrophin expression by DECs after fusion. a** Diagram of ex vivo polyethylene glycol (PEG) mediated cell fusion procedure to create DEC. **b** Flow cytometry plots presenting gating strategy used for DEC analysis; forward (FSC) vs. side (SSC) scatter to remove the signal of cell debris (left plot) and height vs. width side scatter to eliminate cell aggregates (right plot). **c** Representative flow cytometry plots of fusion of the PKH26-labeled MB^*wt*^ and PKH67-labeled MB^*mdx*^ parent myoblasts assessed by FACS. The overlapping fluorescence of PKH26/PKH67 confirms chimeric state for MB^*wt*^/MB^*mdx*^ DEC cell line (far right). **d** Representative immunofluorescence images of dystrophin (magenta) in murine dystrophin-expressing MB^*wt*^, dystrophin-deficient MB^*mdx*^ and MB^*wt*^/MB^*mdx*^ DEC in vitro at 21 days after fusion confirming maintenance of dystrophin expression by DECs (n = 4, magnification 400X, scale bar 10 µm)

After harvesting and counting in 0.4% Trypan Blue staining solution (Gibco-ThermoFischer, Waltham, MA, USA), parent *snj* (*wt)* and *mdx* myoblasts (MB^*wt*^ and MB^*mdx*^) were washed in serum-free DMEM culture media supplemented with 1% Antibiotic-Antimicotic solution (Gibco-ThermoFischer, Waltham, MA, USA). Then, parent myoblasts were fluorescently labeled using either PKH26 (Sigma, St. Louis, MO, USA) or PKH67 (Sigma, St. Louis, MO, USA) tracking membrane dyes according to manufacturer’s instructions. Briefly, each parent cell pellet was suspended in 1 ml diluent C (Sigma, St. Louis, MO, USA) and 6 μl PKH dye was added in 2 ml total volume. After 5-minute room temperature incubation, the staining procedure was stopped with the addition of 1% BSA followed by 1 min incubation and subsequent wash in culture media. Before fusion, parent cells were mixed and washed in serum-free DMEM basal media. After the pellet was re-suspended, the fusion procedure was performed using fusion medium consisting of 3.5 g of polyethylene glycol (PEG 4000, EMD), 400 μl of DMSO (Sigma, St. Louis, MO, USA) and 2 ml of serum-free DMEM basal media supplemented with 1X Antibiotic-Antimicotic solution [27, 28]. The fused cells then were washed in complete culture media and transferred to DPBS-based fluorescently activated cells sorting (FACS) buffer containing 25 mM HEPES, 2 mM EDTA and 1% FBS. Finally, cells presenting double (PKH26/PKH67) staining were selected via FACS (MoFlow Astrios, Beckman Coulter, San Jose, CA, USA) and used for further in vitro analysis or were transplanted to the recipient *mdx* mice. Experimental design is outlined on Fig. 1a. A total of 10 cell fusions were performed to create murine Dystrophin Expressing Chimeric Cells (MB^*wt*^/MB^*mdx*^ DEC) and to characterize DEC in vitro and test efficacy in vivo after intramuscular transplant to *mdx* mice.

### FACS Analysis Confirming DEC Fusion

Following fusion, samples of sorted PKH26/PKH67 labeled DEC, as well as corresponding single stained controls (PKH26 labeled MB^*mdx*^, and PKH67 labeled *snj* MB^*wt*^) and unstained controls (*snj* MB^*wt*^ and MB^*mdx*^) were fixed with 4% paraformaldehyde for 15 min. Samples resuspended in 1X DPBS were analyzed via FACS (Gallios, Beckman Coulter, San Jose, CA, USA).

### Immunofluorescence Detection of Dystrophin Expression In Vitro

Murine DEC (n = 4 fusions) and parent myoblast lines (*snj* MB^*wt*^ and MB^*mdx*,^ n = 4/cell type) were cultured on poly-L-lysin coated German glass coverslips (Corning Inc, New York, USA) placed in 6-well plates (Corning, New York, USA) to confirm dystrophin expression. At 21 days after fusion, cells were fixed with ice-cold acetone, washed, and unspecific antibody binding were blocked with 10% normal goat serum. Mouse monoclonal anti-dystrophin primary antibody (MANDYS8, 1:300, ThermoFischer, Waltham, MA, USA) and goat anti-mouse AlexaFluor-647 conjugated secondary antibody (1:400, ThermoFisher, Waltham, MA, USA) were used for dystrophin detection. Nuclei were counterstained with DAPI (Vector Laboratories, CA, USA). A Zeiss Meta confocal microscope with ZEN software (Carl Zeiss, Oberkochen, Germany) was used for fluorescence signal detection and analysis.

### Proliferation Assay

Cell proliferation of parent myoblasts (*snj* MB^*wt*^ and MB^*mdx*^, n = 4/cell type) before cell fusion procedure and fused DEC (n = 4 fusions/cell type) were monitored by counting in 0.4% Trypan Blue staining solution (Gibco-ThermoFischer, Waltham, MA, USA). Cells were plated and cultured in independent 75 cm^2^ collagen-coated flasks at each time point in F10/DMEM-based Myoblast Growth Medium supplemented with 20% Fetal Bovine Serum (Gemini Bio-products, West Sacramento, CA, USA), 1X Antibiotic-Antimicotic solution (Gibco- ThermoFischer, Waltham, MA, USA) and 50 μl of 25 μg/ml basic fibroblast growth factor (bFGF, Peprotech). Cells were counted on days 0, 3, 6, 9, 12, 15, 18 and 21. Absolute cell count results were normalized by the seeded cell number and expressed as fold increase.

### In Vitro Myogenic Differentiation of Murine DEC

To confirm myogenic differentiation, freshly fused DEC (n = 4 fusions) and parent myoblasts (*snj* MB^*wt*^ and MB^*mdx*^) were cultured on German glass coverslips in serum-free Myogenic Differentiation Medium (Promocell, Heidelberg, Germany) supplemented with 10 μg/ml insulin to induce myogenic differentiation. After seven-day culture, cells were fixed with ice-cold acetone and unspecific antibody binding was blocked with 10% normal goat serum. Rabbit polyclonal anti-myosin heavy chain antibody (1:200, Abcam, Cambridge, MA, USA) was used as primary and goat anti-rabbit Alexa Fluor 647 (1:500, Molecular Probes, OR, USA) conjugated antibody was used as secondary antibody. Nuclei were counterstained with DAPI Vector Laboratories, CA, USA. A Zeiss Meta confocal microscope with ZEN software (Carl Zeiss, Oberkochen, Germany) was used for fluorescence signal detection and analysis.

### Murine DEC Transplantation

The following experimental groups were performed after randomization of age matched 6–8 weeks old *mdx* recipients: vehicle injection (n = 6, 60 μl DPBS), injection of not fused *snj* MB^*wt*^ and MB^*mdx*^ (n = 6, 0.5 × 10^6^ in 60 μl DPBS) and injection of DEC *snj* MB^*wt*^/MB^*mdx*^ (n = 6, 0.5 × 10^6^ in 60 μl DPBS). Cells were counted, washed twice in sterile DPBS and transferred in 60 μl of PBS to tuberculin syringe with 27G needle (Exelint International, Los Angeles, CA, USA) in preparation for intramuscular injection.

*Mdx* recipients were anesthetized with 1.5% isofluorane inhalation and the skin on the left posterior calf was shaved and aseptically prepared. Based on a standard circle shaped template, six microinjections (10 μl/injection, total volume 60 μl) were delivered equidistantly through the skin into the gastrocnemius muscle (GM). Animals recovered in a heated environment and were promptly returned to the colony. The 30-day follow-up included observation of the site of DEC injection animals for presence of ecchymosis, inflammation, or infection. In addition, in vivo muscle strength tests (grip strength and wire hanging) were performed twice a week as described in detail below. At day 30 endpoint, the injected and contralateral untreated GM were harvested for histological and immunofluorescence analysis.

### Histological and Immunofluorescence Analysis of Gastrocnemius Muscle (GM) Cross-Sections

OCT embedded frozen GM muscle was cut with cryotome (ThermoFischer, Waltham, MA, USA) at 4-micron cross-sections, which were fixed with ice-cold acetone. Immuno-blocking was performed with 10% normal goat serum in 1% BSA. Dystrophin was detected using primary anti-dystrophin (1:200, MANDYS8, Abcam, Cambridge, MA, USA) antibody and secondary goat Alexa Fluor (AF) 555 conjugated secondary antibody. Nuclei were counterstained with DAPI Vector Laboratories, CA, USA. A Zeiss Meta confocal microscope with ZEN software (Carl Zeiss, Oberkochen, Germany) was used for fluorescence signal detection and analysis. The number of dystrophin-positive muscle fibers in five standardized regions of each cross-section were counted and normalized to total nuclei numbers; three, non-serial cross-sections were quantified for each animal (n = 6/group).

### Muscle Strength Evaluation

#### Wire Hanging and Grip Strength Tests

Mice motor function was monitored up to 30-day endpoint and animal testing (n = 6/group) was performed in a blinded fashion.

The wire hanging test was repeated a maximum of three consecutive times to prevent animal training for negative performance and the wire hanging time was measured. Although this muscle force evaluation was not specific for the GM, it provided information regarding the general muscle strength of the DEC injected vs. control animals. A modified grip strength test for posterior limbs [29, 30] was used to measure GM-specific force. Briefly, the hook of grip meter (Digital Force Gauge, HL-50) was placed to touch the mouse toes. Upon the presence of grip, the hook was pulled away slowly to assess automatic maximum peak force. Measurements were repeated 10 times and the average maximum peak was used for further analysis.

### Statistical Analysis

All results are presented as mean ± SD. OriginPro 2017 software (OriginLab Corp., Northampton, MA) was used to perform statistical analysis. One-way ANOVA with Tukey’s post-hoc test were used to define statistical significance. P values were considered significant below 0.05.

## Results

### Confirmation of Myoblast Fusion and Creation of Murine Dystrophin Expressing Chimeric Cells (DEC)

To assess the feasibility of PEG mediated cell fusion via flow cytometry, we applied standard gating strategy of forward vs. side scatter to remove the signal of cell debris. Additional correction of height vs. width side scatter was used to eliminate cell aggregates (Fig. 1b). Suitable controls of unstained myoblasts derived from *snj (*MB^*wt*^) and dystrophin deficient mice (MB^*mdx*^) as well as single stain controls of PKH26 labeled *snj* MB^*wt*^ and PKH67 labeled MB^*mdx*^,were used to determine the localization of double stained PKH26/PKH67 cells. The presence of overlapping fluorescence of PKH26/PKH67 indicated the chimeric state for MB^*wt*^/MB^*mdx*^ DEC cell line (Fig. 1c).

### Confirmation of Maintenance of Dystrophin Expression Assessed In Vitro Up to 21 Days After DEC Fusion

To additionally confirm the ex vivo cell fusion feasibility and the potential therapeutic effect of DEC in vivo, we tested in vitro the FACS sorted MB^*wt*^/MB^*mdx*^ DEC for presence of dystrophin expression, a marker of healthy myocytes. We have detected using immunofluorescence staining the maintenance of dystrophin expression in MB^*wt*^/MB^*mdx*^ DEC up to 21 days after fusion. The analysis of immunofluorescence images included the positive (*snj* MB^*wt*^) and negative (dystrophin-deficient MB^*mdx*^) controls to eliminate the false positive results and autofluorescence artifacts (Fig. 1c).

### Confirmation of Proliferative Potential of DEC Up to 21 Days After Fusion

**Fig. 2:**
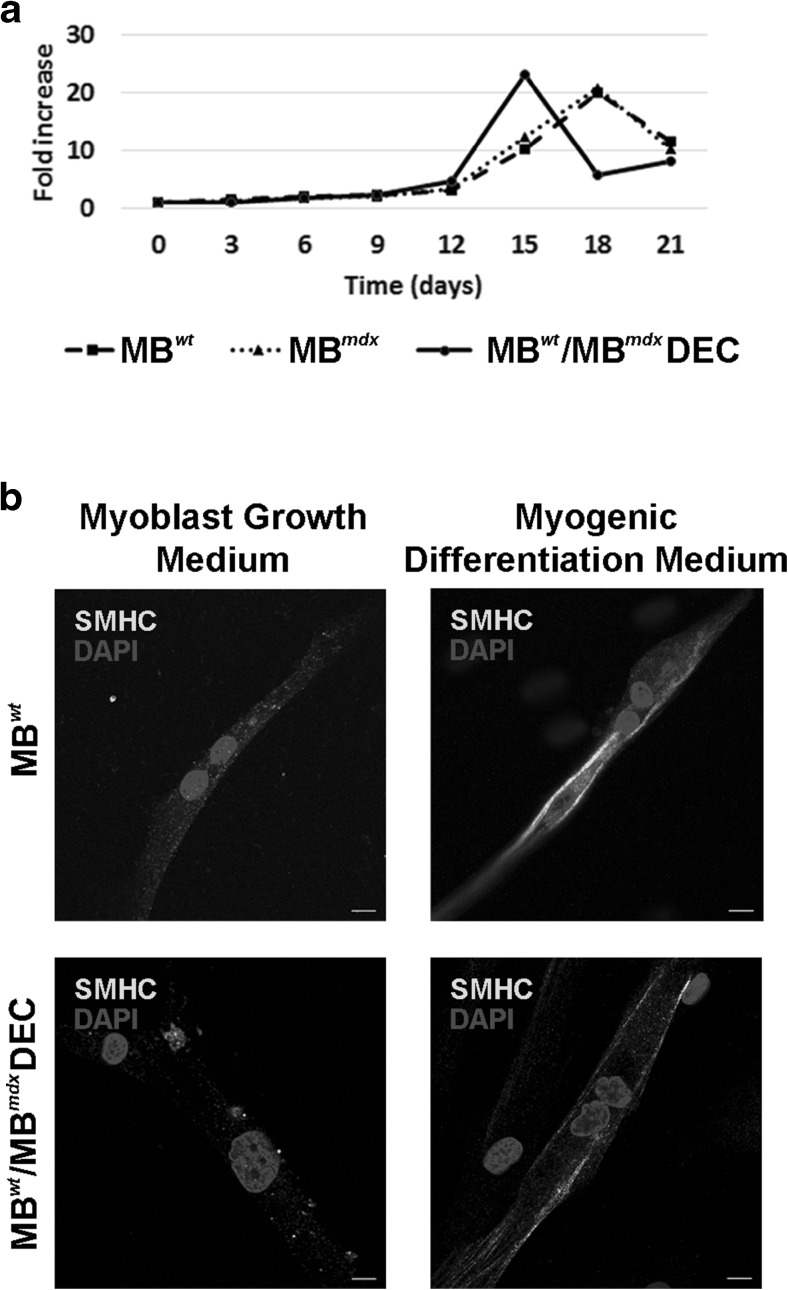
**In vitro analysis of proliferation kinetics and myogenic differentiation of murine Dystrophin Expressing Chimeric Cells (DEC). a** The DEC proliferation kinetics after fusion compared with *snj* wild type myoblasts (MB^*wt*^) and dystrophin deficient *mdx* (MB^*mdx*^) parent cells proliferation up to 21 days of culturing. Absolute cell counts (n = 3/cell type) at each time point (day 0, 3, 6, 9, 12, 15, 18, 21) were normalized with number of seeded cell and proliferation was expressed in fold increase. MB^*wt*^/MB^*mdx*^ DEC cell proliferation results did not show differences in the maximal proliferation counts after fusion when compared to parent myoblast populations (p > 0.05, one-way ANOVA, not significant), confirming maintenance of efficient cell cycle with maximal proliferation peak reached 3 days earlier compared to not-fused controls. **b** Representative immunofluorescence images of DEC differentiated into skeletal myocytes expressing skeletal myosin heavy chain marker (SMHC-AlexaFluor 647, yellow) seven days after fusion. Confirmation of differentiation of MB^*wt*^/MB^*mdx*^ DEC line to the skeletal myocytes in myogenic differentiation media comparable with MB^wt^ controls. For merge: Yellow, SMHC; blue, DAPI (nuclei), scale bar 10 μm

Analysis of proliferation kinetics of *snj* MB^*wt*^ and dystrophin deficient MB^*mdx*^ parent cells and fused DEC after 21 days of in vitro culturing in Myoblast Growth Medium, revealed that the absolute cell counts (n = 3/cell type) normalized for number of seeded cell at each time point (day 0, 3, 6, 9, 12, 15, 18 and 21) and proliferation rate did not show differences in the kinetics of DEC (MB^*wt*^/MB^*mdx*^) cell proliferation as well as in the maximal proliferation counts after fusion when compared to the parent myoblast (MB^*wt*^ and MB^*mdx*^) populations (p > 0.05, one-way ANOVA, not significant). This confirmed maintenance of DEC efficient cell cycle with maximal proliferation peak reached 3 days earlier, at day 15, compared to the peak of not-fused controls reached at day 18 (Fig. 2a).

### Confirmation of Myogenic Differentiation of DEC After Fusion

Analysis of DEC differentiation capacity tested after stimulation in serum-free Myogenic Differentiation Medium (MDM) for seven days after fusion confirmed differentiation of FACS sorted DEC into skeletal myocytes expressing skeletal myosin heavy chain marker (SMHC-AF647, yellow). The increased distribution of SMHC in proximity of sarcolemma was observed in the Myocyte Differentiation Medium cultured MB^*wt*^/MB^*mdx*^ DEC line as well as in the normal *snj* wild type MB^wt^ control (Fig. 2b**)** compared to DEC and MB^wt^ control cultured in Myoblast Growth Medium.

### DEC Significantly Increases Dystrophin Expression at 30 Days After Transplantation to GM of *mdx* Recipient Mouse

**Fig. 3:**
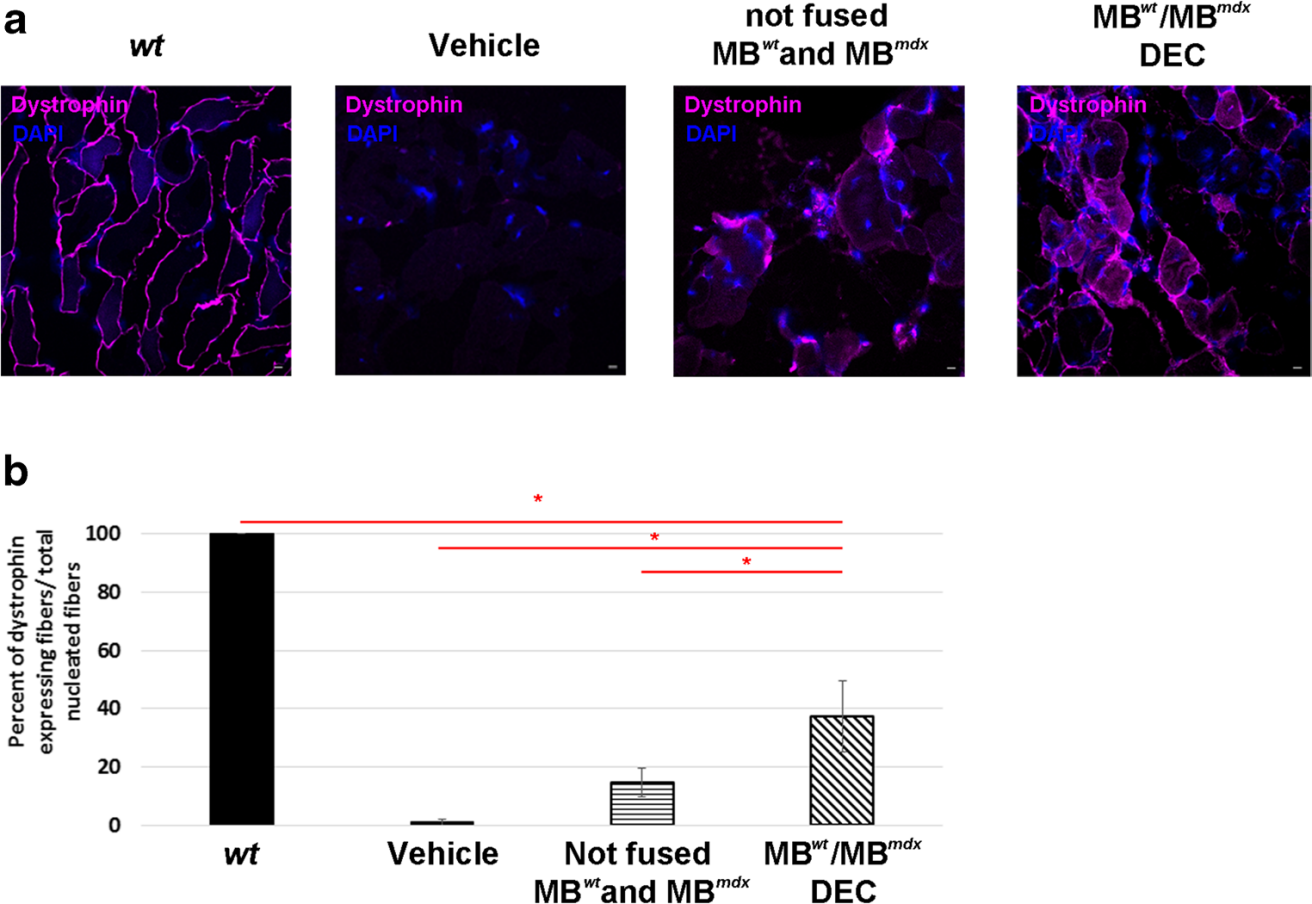
**Significant increase of dystrophin expression at 30 days after DEC transplant to the gastrocnemius muscle (GM) of** ***mdx*** **mice. a** Representative immunofluorescence images of dystrophin expression in GM of *snj* wild type (*wt*) mice (left, positive control), dystrophin-deficient *mdx* mice injected with vehicle, not fused MB^*wt*^ with MB^*mdx*^ and MB^*wt*^/MB^*mdx*^ DEC. Restoration of dystrophin expression (magenta) is confirmed in GM of *mdx* mice injected with DEC. For merge: Magenta, dystrophin; blue, DAPI (nuclei), scale bar 10 μm. **b** Quantification of dystrophin-positive fibers at 30 days post-transplant confirms an increase of 37.27% in DEC injected *mdx* host compared to vehicle and not-fused MB^*wt*^ and MB^*mdx*^ controls; (n = 6, mean ± SD, 5 ROI/3 sections/6 animal/group, One-way ANOVA)

Dystrophin expression was assessed by immunofluorescence staining following myoblasts transplant into the GM of wild type (*wt*) *snj* mice (positive control), dystrophin-deficient *mdx* mice injected with vehicle (negative control), with not fused MB^*wt*^ and MB^*mdx*^ parent cells and with fused DEC (MB^*wt*^/MB^*mdx*^). Immunofluorescence images confirmed restoration of dystrophin expression in GM of *mdx* mice injected with DEC at 30 days post-transplant (Fig. 3a). Quantification of dystrophin expression revealed 37.27 ± 12.1% of positive fibers at 30 days after DEC transplant into the dystrophin -deficient *mdx* host when compared to injection of vehicle and injection of not-fused MB^*wt*^ and MB^*mdx*^ controls (0% and 14.67 ± 5.01%, respectively); (n = 6, mean ± SD, 5 ROI/3 sections/6 animal/group, One-way ANOVA, Fig. 3b). Dystrophin expression correlates with improvement of muscle force at 30 days after DEC transplant to the gastrocnemius muscle of *mdx* mice.

### Functional Outcomes 30 Days After DEC Transplant to the Gastrocnemius Muscles (GM) of *mdx* mice

**Fig. 4:**
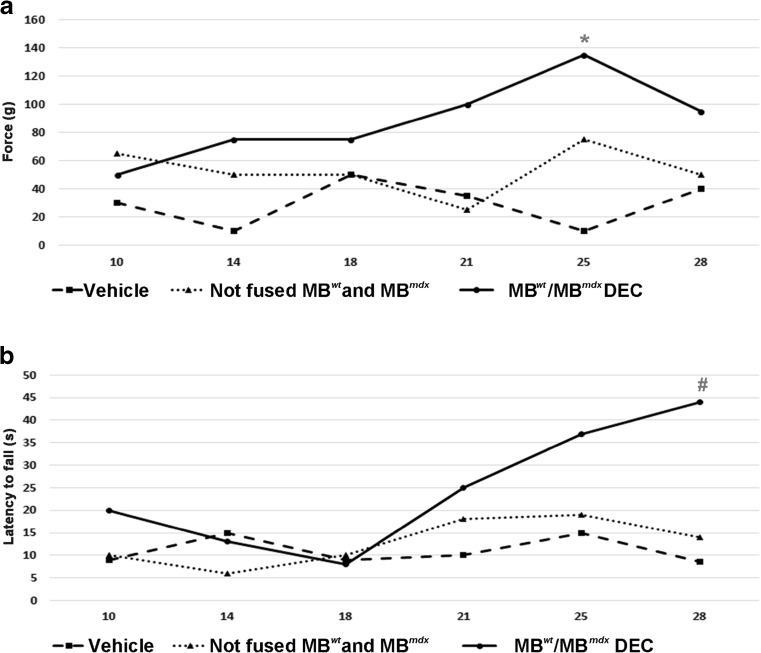
**Improvement of muscle strength and force 30 days after intramuscular DEC transplant to gastrocnemius muscle (GM) of** ***mdx*** **mice. a, b** Representative graphs of grip strength (**a**) and wire hanging test (**b**) of vehicle, not fused MB^*wt*^ and MB^*mdx*^ and fused MB^*wt*^/MB^*mdx*^ DEC injected *mdx *mice (n = 6/group). Mice injected with MB^*wt*^/MB^*mdx*^ DEC improved grip strength and latency to fall (mean, *,^#^ p < 0.05 vs. vehicle, One-way ANOVA). Test results (n = 6/group) were influenced by animal learning and behavioral factors

Standard tests of grip strength and wire hanging tests were assessed in *mdx* mice injected with vehicle, with not fused MB^*wt*^ and MB^*mdx*^ and fused DEC (MB^*wt*^/MB^*mdx*^) (n = 6/group). Mice injected with DEC (MB^*wt*^/MB^*mdx*^) improved grip strength and latency to fall when compared with vehicle injected controls. (mean, *,^#^ p < 0.05, One-way ANOVA). Test results (n = 6/group) were influenced by animal learning and behavioral factors (Fig. 4a, b).

## Discussion

DMD is characterized by progressive muscle wasting and deterioration, which is caused by X-linked mutations of the dystrophin-encoding gene, a structural protein of the cytoskeleton. The lack of dystrophin leads to muscle weakness, degeneration, and consequent fibrosis in skeletal and cardiac muscles [31]. The main objective of the ongoing research is to develop novel and promising strategies to prevent the premature loss of mobility and early mortality of DMD patients through delivery and restoration of dystrophin in affected muscles. Preclinical and clinical approaches in the pipeline include exon skipping, gene editing via viral vectors, and stem cell transplants [2, 3, 32–34]. Stem cell based therapies represent one of the promising approaches for treatment of DMD and have established history of different protocols application in experimental and clinical studies where human myoblasts and bone marrow transplants were tested [12, 33, 35, 36]. Preliminary outcomes of clinical studies were encouraging and have proven safety of human myoblast and hematopoietic stem cell transplantation. However, low efficacy of engraftment due to immune rejection and sub-optimal effects under immunosuppression, resulted in continuous search for new and more effective strategies of stem cell application in DMD [5, 11, 21, 37]. New approaches include transplantation of myogenically converted dermal fibroblasts or genetically modified autologous cells where dystrophin gene is introduced ex vivo by lentivirus prior to transplantation [13, 38]. These studies confirm dystrophin expression and restoration of functional dystrophin following transplantation of the autologous cells engineered to express dystrophin. Most recent strategies propose differentiation of pluripotent stem cells to muscle fibers to modify DMD [39] or application of Embryonic Stem Cells (ESC) - Derived Stromal Cells as a new source of “immortal” human myoblasts which will be produced in abundance for unlimited number of injection sites and will be replicated in vivo without limitations [40]. All these new strategies to engineer, manipulate, or reprogram stem cells to facilitate their engraftment and delivery of functional dystrophin in DMD are both sophisticated and encouraging, however, due to potential side effects of genetic modifications and use of viral vectors, require rigorous pre-clinical testing for both safety and efficacy before clinical application in DMD patients [41]. Moreover, repeated dosing of these therapies may be limited due to sensitization [42]. Transplantation of “immortal” ESC derived myoblast raises another safety issue of the lack of control over the potentially harmful unlimited replication of the transplanted cells. Interestingly, recent clinical reports on myoblast and stem cell transplantation, reveled better engraftment and efficacy and confirmed again the long-term safety of these stem cell based therapies [33].

Thus, encouraged by the ongoing interest in the application of stem cell therapies for DMD and the confirmed long-term safety of myoblast based therapies, we proposed to apply the concept of cell fusion technology to create human chimeric cell lines of myoblast origin as a novel approach for DMD.

Based on our 20 years of experience with tolerance induction and chimerism tested in bone marrow and vascularized composite allotransplantation (VCA) [22–26, 43–47], we proposed to address the limitations of current stem cell therapies, by introducing chimeric cells as a novel stem-cell based approach for DMD.

In this study, we applied the concept on cell fusion to create murine Dystrophin Expressing Chimeric Cells (DEC) via ex vivo fusion of normal myoblast *(snj* MB^*wt*^*)* and dystrophin–deficient myoblast (MB^*mdx*^) using polyethylene glycol (PEG) technique. We have confirmed fusion feasibility and performed in vitro characterization and in vivo assessment of DEC survival, engraftment, and efficacy tested in the *mdx* mouse model of DMD.

Based on the in vitro assessment of DEC in culture, we have confirmed preservation of myogenic differentiation potential and proliferative capacity up to 21 days, which was found to be comparable with normal *snj* MB^*wt*^ controls.

To further test DEC differentiation capacity into myoblast lineage we have analyzed cells after stimulation in myogenic differentiation media for seven days after fusion and confirmed DEC differentiation into skeletal myocytes expressing skeletal myosin heavy chain marker (SMHC-AF647, yellow). Again, differentiation of DEC (MB^*wt*^/MB^*mdx*^) line to the skeletal myocytes was comparable with normal wild type MB^*wt*^ controls.

Previous studies in the *mdx* mouse model showed that in order to trigger muscle force improvement, dystrophin levels should reach 20% [48, 49]. Efficacy studies of exon-skipping therapy conducted by Sharp and others further revealed a linear, dose-dependent therapeutic effect on dystrophin expression in the muscles of *mdx* mice [48].

In our study, therapeutic effect was confirmed after DEC transplant (0.5 × 10^6^) to the gastrocnemius muscle (GM) of the *mdx* mice revealing over 37.27% increase in dystrophin expression, which was almost twofold higher than assessed by others as the level required to elicit functional improvement [48, 49]. Moreover, these results correlated with trends of improved muscle strength and function assessed by wire hanging test and grip strength measurements up to 30-days after DEC transplant, despite the animal learning and behavioral factors [29, 30].

Our approach combines features of clinically established myoblast based therapies, where normal myoblasts with functional dystrophin derived from closely related donor (e.g. father) are transplanted to the DMD-affected son under immunosuppression protocol [36].

Dystrophin expressing chimeric cell therapy represents novel concept of ex vivo fusion of normal and DMD-affected myoblasts to enhance engraftment and minimize or eliminate the need for immunosuppression.

Since DEC therapy is not targeting any specific gene mutation it can be applied to all muscular dystrophies without limitations. This makes DEC therapy attractive and differentiates from other experimental and clinical approaches. The ultimate goal is to bring DEC therapy closer to the clinical application in the DMD patients. To achieve this goal we are currently testing DEC concept by creation of human DEC cell lines with promising preliminary results and expectations to achieve similar functional outcomes as presented in this pilot study on the *mdx* mouse.
